# Codesign of a Quality Improvement Tool for Adults With Prolonged Critical Illness: A Modified Delphi Consensus Study

**DOI:** 10.1097/CCE.0000000000001146

**Published:** 2024-09-12

**Authors:** Laura Allum, Natalie Pattison, Bronwen Connolly, Chloe Apps, Katherine Cowan, Emily Flowers, Nicholas Hart, Louise Rose

**Affiliations:** 1 Florence Nightingale Faculty of Nursing, Midwifery and Palliative Care, King’s College London, London, United Kingdom.; 2 Lane Fox Clinical Respiratory Physiology Research Centre, St Thomas’ Hospital, Guy’s and St. Thomas’ NHS Foundation Trust, London, United Kingdom.; 3 University of Hertfordshire, Hatfield, United Kingdom.; 4 East & North Herts NHS Trust, Stevenage, United Kingdom.; 5 Wellcome-Wolfson Institute for Experimental Medicine, Queen’s University Belfast, Belfast, United Kingdom.; 6 Centre for Human and Applied Physiologic Sciences, King’s College London, London, United Kingdom.; 7 Department of Physiotherapy, The University of Melbourne, Melbourne, VIC, Australia.; 8 Critical Care Research Group and Physiotherapy Department, St. Thomas’ Hospital, Guy’s and St. Thomas’ NHS Foundation Trust, London, United Kingdom.; 9 Katherine Cowan Consulting Limited, East Sussex, United Kingdom.; 10 Physiotherapy Department, King’s College Hospital, London, United Kingdom.; 11 Department of Critical Care and Lane Fox Clinical Respiratory Physiology Research Centre, St Thomas’ Hospital, Guy’s and St. Thomas’ NHS Foundation Trust, London, United Kingdom.

**Keywords:** chronic critical illness, intensive care, prolonged mechanical ventilation, quality improvement

## Abstract

**OBJECTIVES:**

Increasing numbers of patients experience a prolonged stay in intensive care. Yet existing quality improvement (QI) tools used to improve safety and standardize care are not designed for their specific needs. This may result in missed opportunities for care and contribute to worse outcomes. Following an experience-based codesign process, our objective was to build consensus on the most important actionable processes of care for inclusion in a QI tool for adults with prolonged critical illness.

**DESIGN:**

Items were identified from a previous systematic review and interviews with former patients, their care partners, and clinicians. Two rounds of an online modified Delphi survey were undertaken, and participants were asked to rate each item from 1 to 9 in terms of importance for effective care; where 1–3 was not important, 4–6 was important but not critical, and 7–9 was critically important for inclusion in the QI tool. A final consensus meeting was then moderated by an independent facilitator to further discuss and prioritize items.

**SETTING:**

Carried out in the United Kingdom.

**PATIENTS/SUBJECTS:**

Former patients who experienced a stay of over 7 days in intensive care, their family members and ICU staff.

**INTERVENTIONS:**

None.

**MEASUREMENTS AND MAIN RESULTS:**

We recruited 116 participants: 63 healthcare professionals (54%), 45 patients (39%), and eight relatives (7%), to Delphi round 1, and retained 91 (78%) in round 2. Of the 39 items initially identified, 32 were voted “critically important” for inclusion in the QI tool by more than 70% of Delphi participants. These were prioritized further in a consensus meeting with 15 ICU clinicians, four former patients and one family member, and the final QI tool contains 25 items, including promoting patient and family involvement in decisions, providing continuity of care, and structured ventilator weaning and rehabilitation.

**CONCLUSIONS:**

Using experience-based codesign and rigorous consensus-building methods we identified important content for a QI tool for adults with prolonged critical illness. Work is underway to understand tool acceptability and optimum implementation strategies.

**KEY POINTS**

**Questions:** What are the most important actionable processes of care for inclusion in a quality improvement (QI) tool for adults with prolonged critical illness (PCI)?

**Findings:** Thirty-two of 39 included items were voted “critically important” for inclusion in the QI tool by greater than 70% of Delphi participants. These were prioritized further in a consensus meeting: the final QI tool contains 25 items, including promoting patient and family involvement in decisions, providing continuity of care, and structured ventilator weaning and rehabilitation.

**Meanings:** Using experience-based codesign and rigorous consensus-building methods, we identified important patient-centered content for a QI tool for adults with PCI.

Patients experiencing a prolonged ICU stay (i.e., > 7 d) are more likely to encounter symptoms of post-intensive care syndrome (1, 2). They are often receiving no or minimal sedation, so are awake and may be distressed, uncomfortable, and bored (3–5). Their relatives are more likely to experience poor mental health and financial difficulty given this prolonged exposure to ICU (6, 7). Prolonged stays comprise 9% of admissions but 45% of ICU bed days (8), so are expensive for healthcare systems with subsequent prolonged hospital stays outside ICU and ongoing community input once discharged from hospital (9, 10).

Patients experiencing a prolonged ICU stay no longer require resuscitation and stabilization and instead need patient-centered interprofessional strategies that promote rehabilitation and recovery (11). Quality improvement (QI) tools used to improve safety and standardize care for acutely critically ill patients (12, 13) may not therefore equate to high-value care for patients experiencing a prolonged critical illness because of their differing needs. Commonly used tools such as ABCDEF have not been validated in the long-stay patient population (14). Our previous scoping review identified no tools designed to coordinate the overall care of prolonged stay patients (15), and clinicians report a lack of confidence in their management (16). This gap has implications on patient outcomes as development of prolonged critical illness may be inversely related to weaning protocol use and effective interprofessional working (1).

This study is part of a research program aiming to improve the quality of care for patients experiencing a prolonged ICU stay. We have used experience-based codesign methods to identify actionable processes of care (interventions carried out by clinicians at the bedside) of importance to this patient group, their families and the clinicians who care for them (11, 16, 17). The aim of this current study was to obtain consensus on those actionable processes identified through our codesign methods that should be included in a QI tool. Similar work has been carried out in Canada with a resultant published tool (18). This article adds an international comparison given differing health systems and practices including differing models of restraint use (19) and family involvement (20).

## METHODS

### Study Design

Using principles of experience-based codesign (21) and participatory research (22), we conducted a modified Delphi consensus study (23), with the content of the first Delphi round developed from our previous systematic review (11) and exploratory qualitative interviews with patients and family members (17), and with clinicians (16) representing the ICU interprofessional team.

### Ethical Considerations

We obtained research ethics approval from the London—Southeast Research Ethics Committee, reference 19/LO/0328 in May 2019, as part of a wider research project Identification: 225003, “Actionable processes of care for persistent critical illness.” The study was carried out in accordance with the ethical standards of the responsible committee on human experimentation (institutional or regional) and with the Helsinki Declaration of 1975. Participation in the Delphi survey was considered indicative of informed consent. All participants attending the consensus meeting provided written informed consent.

### Participants

We recruited participants to three stakeholder groups, that is, patients, family members, and ICU clinicians. We used a range of strategies, recruiting clinicians via recruitment flyers posted by U.K. professional societies (e.g., British Association of Intensive Care Nurses, Association of Chartered Physiotherapists in Respiratory Care), and patients and families via the U.K. ICU patient charity ICUSteps, social media (X) accounts; emails directed to patients attending an ICU recovery clinic of a large critical care service (approximately 80 beds) in a tertiary academic center in central London, United Kingdom; and snowballing methods (24).

We used a purposive sampling strategy (25) to achieve variation in clinician profession for both our modified Delphi study and subsequent consensus meeting. We recruited nurses, intensivists, speech and language therapists, occupational therapists, psychologists, physiotherapists, dietitians, and pharmacists. We used convenience sampling to recruit former patients and family members. Eligibility criteria included adults over 18 years old and with an ICU stay of more than 7 days within the last 2 years.

### Questionnaire Design

The actionable processes of care for inclusion in round 1 of the Delphi study identified through our item generation work were reviewed by the research team to ensure no redundancy and to remove any items outside project scope. We consulted our advisory group comprising 13 clinicians (two intensivists, two physiotherapists, two Occupational Therapists, two Speech and Language Therapists, three nurses, one pharmacist, and one dietitian), two former patients, and one relative to help us with the lay descriptions for each item and to confirm clarity of wording. This resulted in inclusion of 39 actionable processes of care in round 1.

### Data Collection

Delphi rounds were administered using DelphiManager software (Version 5; Core Outcome Measures in Effectiveness Trials initiative, Liverpool, United Kingdom). In round 1, participants were asked to rate importance of each actionable process of care for inclusion in a QI tool. Participants were provided with a Likert scale with scoring as follows: 1–3 (not important), 4–6 (important but not critical), and 7–9 (critically important) as recommended by the Grading of Recommendations, Assessment, Development, and Evaluation (GRADE) working group (26). An “unable to score” option was provided. Participants were also invited to suggest additional actionable processes of care. Following the completion of round 1, the research team reviewed these additional processes of care to remove duplicates or those beyond the project scope. This included processes that were not actionable at the bedside or focused on care after ICU discharge. In round 2, participants were provided with their own round 1 ratings, the ratings of the other two stakeholder groups, and asked to rerate each actionable process.

We had planned to discuss all items rated critically important (i.e., > 7) by more than 70% of Delphi participants in the consensus meeting to reach a consensus on which should be included in the QI tool. However, this decision was revised on review of round 2 results, which would have required discussion of too many items in the consensus meeting. We, therefore, made a pragmatic decision to discuss at the consensus meeting only those items voted with a mean score of greater than or equal to 7 but less than 8 or those items voted as critical for inclusion by one stakeholder group but less important by others. All items rated greater than 8 were automatically included in the tool.

The consensus-building workshop was held and digitally recorded using Zoom and drew methodologically on the James Lind Alliance method (27). It was facilitated by an independent and experienced facilitator (K.C.) to optimize inclusion of all voices in the meeting. Before the meeting, all participants were sent detailed information packs comprised of a summary of the project (including the 20 items of care where consensus had already been achieved), meeting procedures, and brief participants biographies written by participants to share with others. They also received a worksheet containing a list of 18 actionable processes of care where consensus was not achieved through the modified Delphi (i.e., > 7 and < 8 or not by others), which they were asked to review and rank in order of importance before the meeting. The meeting consisted of an initial briefing then two rounds of small group discussions, where all participants (five in each group with a mix of participants, including one patient in each group) were given equal time to discuss their three highest- and three lowest-priority items. This process was repeated in a second round of small group discussions with people moved into new groups to ensure an exchange of different perspectives. All participants then voted anonymously on the inclusion of actionable processes for the QI tool one final time using a Qualtrics Seattle, WA survey (https://www.qualtrics.com), rating them critical, important but not critical, or not important to include. The results of this vote were shared with the group before the meeting closed, to demonstrate the results of the work to participants.

### Data Analysis

For each Delphi survey and consensus discussion round, we calculated the proportion of participants rating an item as critical for inclusion, important but not critical, and not important. We also calculated the overall mean and sd score and separately for each of the three stakeholder groups. Items rated with a mean score of over 8 by all stakeholder groups were automatically included in the QI tool and not discussed in the consensus meeting. Those items were rated between 7 and 8 or voted as critical by one, but not all stakeholder groups were taken forward for consensus discussion.

## RESULTS

We recruited 116 participants to the first Delphi round, including 45 (39%) former ICU patients and 8 (7%) family members. Most (73%) participants were female, 53% were based in London and the Southeast of England, and 89% were White (**Table 1**). Of the 116 participants, 91 (78%) were retained in round 2 of the Delphi. Further information can be found in **Supplementary Table 1** (http://links.lww.com/CCX/B393).

**TABLE 1.** Participant Demographics

Demographic Category	Round 1, *n* =116, *n* (%)	Round 2, *n* = 91, *n* (%)
Participant type		
Clinician	63 (54)	49 (54)
Patient	45 (39)	38 (42)
Family	8 (7)	4 (4)
Sex		
Female	73 (63)	55 (60)
Male	41 (35)	35 (38)
Other/prefer not to say	2 (2)	1 (1)
U.K. location		
London/Southeast	62 (53)	47 (52)
Northwest England	10 (9)	9 (10)
East of England	9 (8)	9 (10)
Southwest England	9 (8)	7 (8)
Wales	9 (8)	7 (8)
East/West Midlands	8 (7)	6 (7)
Yorkshire and the Humber	4 (3)	2 (2)
Northern Ireland	2 (2)	2 (2)
Northeast England	2 (2)	1 (1)
Scotland	1 (1)	1 (1)
Ethnicity		
White	103 (89)	79 (87)
Asian or Asian British	4 (3)	4 (4)
Mixed or Multiple ethnic groups	2 (2)	2 (2)
Black, Black British, Caribbean, or African	2 (2)	2 (2)
Other	5 (4)	4 (4)

Of the 39 items provided in Delphi round 1, 29 were voted as critically important by more than 70% of participants. Participants suggested 61 additional items. However, after team discussions no new items were added to round 2 as those suggested were deemed either not actionable or out of scope (**Supplementary Table 2**, http://links.lww.com/CCX/B393).

In round 2, 32 items were voted critically important for inclusion by more than 70% of participants. Two items were voted critical for inclusion by all 116 participants. These were “assess and manage symptoms (i.e., pain, breathlessness, tiredness, thirst)” and “regular physical rehabilitation (including early mobilization) with setting and assessment of progress on weekly rehabilitation goals.”

There were 18 items put forward for consensus discussion that had a mean score of greater than or equal to 7 but less than or equal to 8, or alternatively with a mean score of greater than 8 by only one stakeholder group (**Supplementary Table 3**, http://links.lww.com/CCX/B393). A further 20 items were automatically included in the QI tool given they had a mean score greater than 8 across all stakeholder groups. The lowest-rated item was “limit physiologic monitoring and routine blood tests” (voted critical for inclusion by just 18% of participants) and was the only item eliminated at this stage.

We recruited 20 participants (15 clinicians, four former patients, and one family member) to the consensus-building workshop. Of the 18 actionable processes of care discussed, four items were unanimously voted for inclusion (“Appropriate and timely discharge planning (discuss and arrange safe transitions in care location), Conduct interprofessional team meetings to discuss patient- and family-centered care plan, Provide activities to promote cognitive stimulation based on patient preferences, and Provide regular proactive family meetings to set goals; devise a care plan; and share information”) (**Table 2**). A further four items were not unanimous and were voted critical by one group but not by the other. Of these four, one item (“Use a patient diary to aid communication with and provide psychological support for patient and family”) was included because of the evidence base for diary use in U.K. ICUs. Another item (“Preparing patient for more independence where possible; for example, encouraging self-care activities; reducing observations [may require referral to occupational therapy]”) was discarded by participants. The final two items merged with other already included items on the suggestion of participants, who also suggested merging other items (**Table 3**).

**TABLE 2.** Consensus-Building Workshop Results

Item	% Voting Item Critical		Include/Exclude From Tool
	Patients and Family	Staff	
A: Appropriate and timely discharge planning (discuss and arrange safe transitions in care location)	3 (60)	12 (80)	Include
D: Conduct interprofessional team meetings to discuss patient- and family-centered care plan	4 (80)	14 (93)	
O: Provide activities to promote cognitive stimulation based on patient preferences	3 (60)	9 (60)	
P: Provide regular proactive family meetings to set goals, devise a care plan, and share information	3 (60)	13 (87)	
B: Assess endocrine function and treat endocrine dysfunction such as hyperglycemia and hypothyroidism	0 (0)	0 (0)	Exclude
C: Assess/prevent ocular disorders arising from incomplete eyelid closure	1 (20)	0 (0)	
E: De-escalate (including change to oral instead of IV drugs) or stop ICU pharmacotherapy and restart previous comorbidity pharmacotherapy	1 (20)	5 (33)	
F: Enable access to activities (radio, television, iPad) and personal possessions (including clothes) to prevent boredom, loneliness, and restore normality	2 (40)	8 (53)^a^	
G: Enable continuity of care using shared interprofessional goals agreed with family and patient where possible	2 (40)	14 (93)^a^	
H: Ensure access to outside space where possible	1 (20)	4 (27)	
I: Family participation in care and occupation tasks	1 (20)	1 (7)	
J: Family presence or participation in rounds and planning meetings	2 (40)	1 (7)	
K: Include the patient (when able) and family in the development of the weaning plan	0 (0)	1 (7)	
L: minimizing visiting restrictions	1 (20)	2 (13)	
M: Preparing patient for more independence where possible, for example, encouraging self-care activities, and reducing observations (may require referral to occupational therapy)	2 (40)	9 (60)^a^	
N: Provide access to social support such as a social worker or signposting to sources of support and advice agencies (patient and family)	2 (40)	3 (20)	
Q: Use a structured tool (i.e., weaning protocol or individualized weaning plan) to plan and guide weaning developed by the ICU team	1 (20)	6 (40)	
R: Use a patient diary to aid communication with and provide psychologic support for patient and family	3 (60%)^a^	6 (40)	

a An item rated critical by > 50% of one group but not the other.

**TABLE 3.** Merging of Actionable Processes of Care

Original Actionable Process of Care	Merged Item
Enable continuity of care using shared interprofessional goals agreed with family and patient where possible	Conduct interprofessional team meetings to discuss patient- and family-centered care plan with interprofessional goals
Conduct interprofessional team meetings to discuss patient- and family-centered care plan	
Assess and track ventilator weaning progress	Track and guide ventilator weaning using a structured tool (protocol/individualized weaning plan)
Use a structured tool (i.e., weaning protocol or individualized weaning plan) to plan and guide weaning developed by the ICU team	
Assess and treat respiratory muscle weakness	Assess and treat causes of weaning failure, including respiratory muscle weakness and endocrine function
Assess endocrine function and treat endocrine dysfunction such as hyperglycemia and hypothyroidism	
Provide activities to promote cognitive stimulation based on patient preferences	Enable access to activities (radio, tv, iPad) and personal possessions (including clothes) to prevent boredom, delirium, loneliness, and restore normality—might require a referral to Occupational Therapy
Enable access to activities (radio, tv, iPad) and personal possessions (including clothes) to prevent boredom, loneliness, and restore normality	
Identify and use patient preferences for strategies to promote sleep	Use patient preferences for strategies to promote sleep, including reducing nighttime light/noise
Minimize practices such as nighttime light/noise that promote delirium	

tv = television.

**Figure 1.**
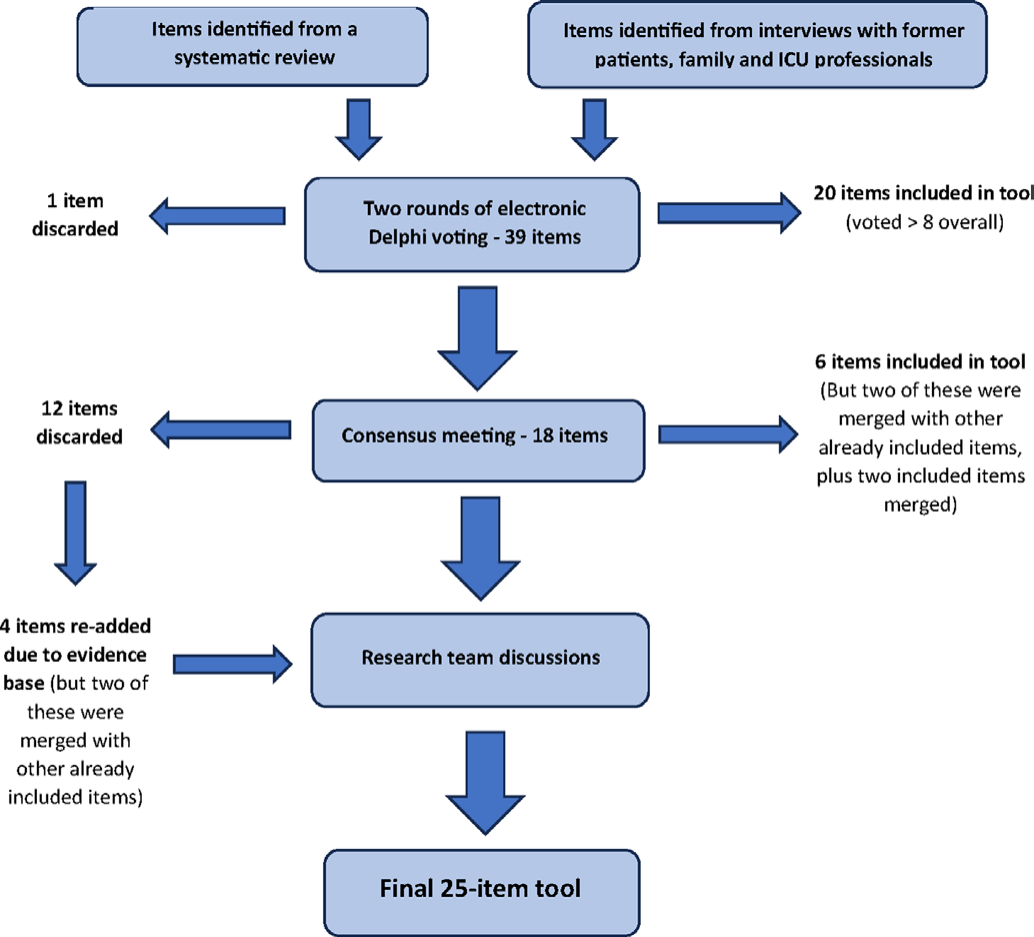
A flowsheet showing the decision-making process for included items.

Analysis of consensus meeting group discussions revealed reasons behind voting decisions. For example, clinician participants were strongly opposed to family presence during rounds, feeling it would make rounds too long and impede teaching opportunities for trainees. Three further items were excluded by Consensus workshop participants because they regarded them as already well-established processes of care (“Assess endocrine function and treat endocrine dysfunction such as hyperglycemia and hypothyroidism”; “De-escalate [including change to oral instead of IV drugs] or stop ICU pharmacotherapy and restart previous comorbidity pharmacotherapy”; and “Use a structured tool (i.e., weaning protocol or individualized weaning plan) to plan and guide weaning developed by the ICU team”). Subsequently, the research team searched for evidence supporting the assumptions of the workshop participants. Contrary to this assumption, evidence was found that they are not implemented consistently (28–30) and may contribute to the persisting critical illness (1, 31). Therefore, these items were included in the tool. This process is described in **Figure 1** below and in **Supplementary Figure 1** (http://links.lww.com/CCX/B393).

Two items were not included in the tool despite meeting the 70% threshold for inclusion in the Delphi because they were voted out in the consensus-building stage: “Assess/prevent ocular disorders arising from incomplete eyelid closure” and “Preparing patient for more independence where possible, for example, encouraging self-care activities, reducing observations (may require referral to occupational therapy).” Participants felt that ocular disorder prevention was more applicable to the acute phase of care and that preparing patients for more independence was inherent in the aims of physical and occupational rehabilitation.

**Figure 2.**
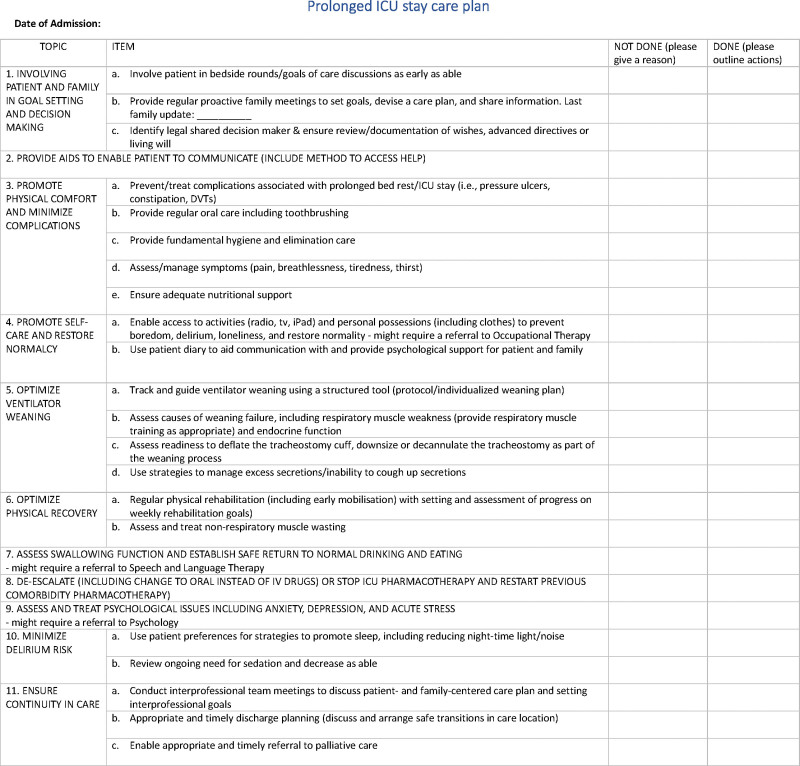
The final prolonged ICU stay care plan. DVT = deep vein thrombosis, tv = television.

The final list for inclusion in a QI tool comprised 25 actionable processes for inclusion in a QI tool (**Fig. 2**).

## DISCUSSION

Using rigorous item generation and consensus methods we identified 25 actionable processes for inclusion in a QI tool for patients experiencing a prolonged ICU stay. Our results demonstrate the complexity of care required for this patient group; with 32 of 39 items identified from a review of the evidence base (11) and interviews with patients, family members, and clinicians (16, 17) voted as critically important by more than 70% of participants. This testifies to the consequences of missing these important care activities (15, 32) for this patient group.

Items promoting comfort and recovery received the highest proportions of “critical importance” votes. This likely reflects the frequency of poorly controlled symptoms in ICU patients (33–36) and significant physical impact of a prolonged ICU stay (3, 37). Many other prioritized items relate to what has been termed “humanization of care” (38–40) including the promotion of psychologic wellbeing, a safe return to eating and drinking, regular washes and dignified toileting, and effective methods of communication for patients unable to speak. This speaks to the distinction between the needs of the acutely unwell patient who primarily needs highly medicalized care and the longer-term patient who may be awake and requiring reassurance and rehabilitation.

Interestingly, although items relating to family support were rated important or critically important in the Delphi, our participants did not prioritize these items highly enough for their inclusion in the tool. This includes family participation in care, family presence in rounds and planning meetings, and minimizing visiting restrictions. This is surprising given the importance of these activities for family wellbeing, patient recovery and concordance between family members and the ICU team (41–43), and reflects a lack of prioritization of family needs. Although 100% of family members voted family presence on rounds as critically important only 62% of patients and 35% of healthcare professionals agreed. In the United Kingdom, it is not standard practice for family members to be present during ICU rounds as it is in Canada. Our participants were also in favor of visiting restrictions, feeling that patients and staff should have time without visitors for rest and for healthcare professionals to catch up on tasks despite evidence that this is worse for family members’ mental health outcomes (44). Our participants also did not prioritize the need for signposting families to sources of information and a social worker. This is despite the well-documented impact on family members of a prolonged ICU stay (45) and evidence suggesting information provision can reduce anxiety and post-traumatic stress disorder (46, 47). This finding may also have been impacted by the low numbers of family members taking part, but while family members gave these items more importance than patients and clinicians, they still voted these items in the lowest third of mean scores (Supplementary Table 2, http://links.lww.com/CCX/B393). It is possible that when pushed to choose, participants prioritized the needs of the unwell patient over their family member, as is commonly seen in the ICU setting (6). We acknowledge that the lack of family-related items in this tool is antithetical to the evidence base and widely accepted tools such as the ABCDEF bundle. In our future work assessing the feasibility and impact of this tool, we plan to capture satisfaction and qualitative data from family members to determine the impact of this omission.

We include six items in the tool despite being voted out by consensus-building participants. When the research team reviewed the discussions, it became obvious that these items were excluded as believed to be implemented widely in U.K. ICUs. The evidence base, however, indicates these practices are not implemented consistently; resulting in inconsistent deprescribing on ICU discharge (30, 48), use of structured weaning tools (29, 49), and ICU diaries (28) and risking further prolongation of critical illness (1). Therefore, the decision was taken to add these back in.

There is an argument for including more items in our tool, given that almost all items were rated critically important by most participants. However, we were keen to create a usable tool and recognize that the time required to complete the tool can act as a barrier to its use (50, 51). This is particularly important in current health pressures (52–54) and with poor ICU staff morale (5, 55). Our group previously created a QI tool using the same methods with Canadian participants (18). These tools are similar in content but with some differences. The Canadian tool has a greater focus on family support, with inclusion of family on ICU rounds and in the development of a weaning plan. It also includes minimizing physical restraints. Physical restraint is much more prevalent in Canadian practice (19, 56), and it is not typical for family members to attend rounds in the United Kingdom. In contrast to their Canadian counterparts, U.K. participants voted for the inclusion of ICU diaries, which are commonly used in U.K. practice and not in Canada. U.K. participants also prioritized early and communicated discharge planning, possibly reflecting differences in funding models of home-based care between the countries, and interprofessional meetings to improve care continuity.

Strengths of our study include rigorous use of experience-based codesign and consensus methods informing study design, recruitment, and data collection processes with inclusion of patients, family members, and clinicians. We had strong patient representation at every stage of the study.

Limitations of our study include a low number of family members (eight in first Delphi round and one for the consensus-building stage). Despite specific recruitment methods targeting family members, we were unable to improve these numbers. Our survey population lacked diversity in terms of ethnicity (89% of participants were White in the first Delphi round) and regional representation (62% from London and Southeast England, as opposed to 12% from Scotland, Wales, and Northern Ireland combined). Again, we had made specific attempts to improve this diversity, including approaching organizations that aim to improve patient and family participation in research (Healthwatch England, healthwatch.co.uk UK, ICUSteps) and using U.K.-wide organizations to distribute recruitment materials. We did not collect data describing the ICU stay of our patient participants and acknowledge the heterogeneity of prolonged stays (with predictably long stays with conditions like Guillain-Barré compared with persistently critically ill patients with recurrent episodes of sepsis), so we cannot know whether we captured a full range of prolonged stay experiences.

Because so many items were voted of critical importance by participants in our modified Delphi survey, we also made a pragmatic choice to include those items rated greater than 8 without further discussion but discuss those voted between 7 and 8 in a consensus meeting. GRADE criteria suggest items voted greater than 7 should be considered for inclusion, so these criteria were followed albeit with a pragmatic decision not to discuss further the most highly rated items.

Last, we acknowledge that the number of items in the tool may make it difficult to implement in practice. We have since undertaken work exploring tool feasibility in a single London hospital, which we plan to publish separately.

## CONCLUSIONS

Using rigorous methods and informed by all relevant stakeholders, we developed a 25-item QI tool for use with patients experiencing a prolonged stay in ICU. We anticipate this QI tool will aid in the standardization of care with the potential to prevent errors of omission constituting inadequate care and contributing to negative patient outcomes. We propose that this tool could be used at a once or twice-weekly interprofessional meeting held at the patient’s bedside. This would enable each item on the tool to be discussed with the patient and/or family members. This would promote communication consistency and patient and family agency in their care plan.

Further work is now needed to understand elements of tool implementation including acceptability and feasibility.

## Supplementary Material
