# Supplementary material for: Codesign of a Quality Improvement Tool for Adults With Prolonged Critical Illness: A Modified Delphi Consensus Study
Source: Crit Care Explor. 2024 Sep 12;6(9):e1146. doi: 10.1097/CCE.0000000000001146 (PMC11390055; doi:10.1097/CCE.0000000000001146)
Supplement: Supplementary file 1 [file cc9-6-e1146-s001.pdf]

Supplementary material: Table 1

CLINICIANS ONLY<br>Round 1 = 55<br>Round 2 = 45	Profession	Physiotherapist	15	14
		Nurse	14	9
		Dietitian	12	8
		Doctor	7	7
		Occupational Therapist	5	3
		Speech and Language Therapist	5	5
		Pharmacist	3	2
		Psychologist	1	0
		Other (not stated)	1	1
	Years of clinical experience	Less than 5 years	20	15
		5-10 years	11	8
		More than 10 years	32	26
Patient and family<br>Round 1 = 53<br>Round 2 = 42	Age	18-30	5	3
		31-45	12	9
		46-55	10	9
		56-65	18	13
		66-75	6	6
		Over 75	2	2
	Recency of ICU stay	Within last 2 years	24	18
		2-5 years ago	19	10
		More than 5 years	10	14
	Length of stay	7-14 days	13	8
		15-31 days	22	20
		1-2 months	12	9
		3-6 months	6	5
Family only<br>Round 1 = 8<br>Round 2 = 4	Relationship to patient	Son or daughter	2	1
		Sibling	2	0
		Parent	2	2
		Partner	2	1

Table 2 Delphi Survey: Proportions voting critical

ACTIONABLE PROCESS OF CARE	ROUND ONE PROPORTIONS VOTING CRITICAL (%)				ROUND TWO PROPORTIONS VOTING CRITICAL (%)			
	Overall	Staff	Family	Patients	Overall	Staff	Family	Patients
Assess and manage symptoms (i.e., pain, breathlessness, tiredness, thirst)	103/114 (90)	59/63 (94)	8/8 (100)	36/43 (84)	89/89 (100)	49/49 (100)	4/4 (100)	36/36 (100)
Regular physical rehabilitation (including early mobilisation) with setting and assessment of progress on weekly rehabilitation goals	107/113 (95)	60/62 (97)	8/8 (10)	39/43 (91)	87/87 (100)	48/48 (100)	3/3 (100)	36/36 (100)
Assess and treat non-respiratory muscle wasting	100/113 (89)	54/63 (86)	8/8 (100)	38/42 (90)	85/86 (99)	47/48 (98)	3/3 (100)	35/35 (100)
Use strategies to manage excess secretions or the inability to cough up secretions	106/110 (96)	59/60 (98)	8/8 (100)	39/42 (93)	85/86 (99)	47/47 (100)	4/4 (100)	34/35 (97)
Assess and treat psychological issues including anxiety, depression, and acute stress (with referral to psychiatry if required)	101/114 (89)	53/62 (85)	7/8 (88)	41/44 (93)	85/87 (98)	46/48 (96)	4/4 (100)	35/35 (100)
Ensure adequate nutritional support	106/114 (93)	62/63 (98)	8/8 (100)	36/43 (84)	85/87 (98)	48/48 (100)	3/3 (100)	34/36 (94)
Provide fundamental hygiene and elimination care	103/116 (89)	59/63 (94)	8/8 (100)	36/45 (80)	85/87 (98)	47/47 (100)	3/3 (100)	35/37 (95)
Assess readiness to deflate the tracheostomy cuff, downsize or decannulate the tracheostomy as part of the weaning process	88/95 (93)	55/57 (96)	6/6 (100)	27/32 (84)	73/76 (96)	46/47 (98)	2/2 (100)	25/27 (93)
Assess and treat respiratory muscle weakness	89/109 (82)	46/60 (77)	8/8 (100)	35/41 (85)	81/85 (95)	44/47 (94)	3/3 (100)	34/35 (97)
Assess swallowing function and establish safe return to normal drinking and eating (may require referral to speech and language therapy)	103/113 (91)	59/63 (94)	7/8 (88)	37/42 (88)	81/85 (95)	48/48 (100)	2/3 (67)	31/34 (91)
Enable appropriate and timely referral to palliative care	78/93 (84)	52/62 (84)	5/5 (100)	21/26 (80)	69/73 (95)	45/47 (96)	2/2 (100)	22/24 (92)
Provide aids to enable patients to communicate (including method to access help) (referral to speech specialist if required)	92/114 (81)	59 /63 (94)	8/8 (100)	25/34 (74)	79/83 (95)	49/49 (100)	4/4 (100)	31/35 (89)
Assess, prevent/treat complications associated with prolonged bed rest/ICU stay (i.e., pressure ulcers, constipation, DVTs)	102/116 (88)	61/64 (95)	7/8 (88)	34/44 (77)	83/88 (94)	47/48 (98)	3/3 (100)	33/37 (89)
Identify legal shared decision maker & ensure review/documentation of wishes, advanced directives or living will	80/97 (82)	54/63 (86)	5/6 (83)	21/28 (75)	73/78 (94)	46/48 (96)	2/3 (67)	25/27 (93)
Assess and track ventilator weaning progress	94/105 (90)	56/60 (93)	5/6 (83)	33/39 (85)	77/83 (93)	42/45 (93)	2/3 (67)	31/33 (94)
Minimize practices such as night-time light/noise that promote delirium	89/113 (79)	53/63 (84)	5/8 (63)	31/42 (74)	81/87 (93)	48/49 (98)	4/4 (100)	29/34 (85)
Assess/prevent ocular disorders arising from incomplete eyelid closure	80/98 (82)	52/61 (85)	7/7 (100)	21/30 (70)	69/75 (92)	45/46 (98)	2/2 (100)	22/27 (81)
Review ongoing need for drugs used for sedation and decrease their use as able	91/108 (84)	58/62 (94)	7/7 (100)	26/39 (67)	78/85 (92)	48/49 (98)	3/3 (100)	27/33 (82)
Appropriate and timely discharge planning (discuss and arrange safe transitions in care location)	94/112 (84)	54/63 (86)	6/8 (75)	34/41 (83)	79/87 (91)	44/48 (92)	3/3 (100)	32/36 (89)
Provide regular oral care including toothbrushing	99/116 (85)	60/63 (95)	8/8 (100)	31/45 (69)	79/87 (91)	47/47 (100)	3/3 (100)	29/37 (78)

Assess endocrine function and treat endocrine dysfunction such as hyperglycemia and hypothyroidism	72/92 (78)	45/58 (78)	6/6 (100)	21/28 (75)	64/71 (90)	42/45 (93)	2/2 (100)	20/24 (83)
Identify and use patient preferences for strategies to promote sleep	84/111 (76)	48/62 (77)	8/8 (100)	28/41 (68)	76/87 (87)	45/49 (92)	3/3 (100)	28/35 (80)
Involve the patient in bedside rounds and goals of care discussions as early as able	88/114 (77)	51/63 (81)	8/8 (100)	29/43 (67)	77/89 (87)	42/49 (86)	4/4 (100)	31/36 (86)
Enable continuity of care using shared interprofessional goals agreed with family and patient where possible	86/113 (76)	48/63 (76)	6/8 (75)	32/42 (76)	72/87 (83)	40/48 (83)	3/3 (100)	29/36 (81)
Provide regular proactive family meetings to set goals, devise a care plan, and share information	84/114 (74)	47/63 (75)	8/8 (100)	30/43 (70)	70/84 (83)	41/48 (85)	3/3 (100)	26/33 (79)
Use a structured tool (i.e. weaning protocol or individualized weaning plan) to plan and guide weaning developed by the ICU team	76/99 (77)	50/58 (86)	2/5 (40)	24/35 (69)	69/83 (83)	43/47(91)	0/3 (0)	26/33 (79)
Conduct interprofessional team meetings to discuss patient and family-centered care plan	84/114 (74)	50/63 (79)	5/8 (63)	29/43 (67)	70/87 (80)	39/48 (81)	3/3 (100)	28/36 (78)
Preparing patient for more independence where possible, for example encouraging self-care activities, reducing observations (may require referral to occupational therapy)	82/114 (72)	51/63 (81)	4/8 (50)	27/43 (63)	67/86 (78)	39/47 (83)	2/3 (67)	26/36 (72)
Use patient diary to aid communication with and provide psychological support for patient and family	79/111 (71)	40/63 (63)	5/8 (63)	34/40 (85)	65/87 (75)	34/49 (69)	2/4 (50)	29/34 (85)
Enable access to activities (radio, tv, iPad) and personal possessions (including clothes) to prevent boredom, loneliness, and restore normality	74/115 (64)	52/64 (81)	5/8 (63)	17/43 (40)	64/87 (74)	41/48 (85)	2/3 (67)	21/36 (58)
De-escalate (including change to oral instead of IV drugs) or stop ICU pharmacotherapy and restart previous comorbidity pharmacotherapy	70/101 (69)	43/60 (72)	5/7 (71)	22/34 (65)	59/80 (74)	34/46 (74)	2/2 (100)	23/32 (72)
Provide activities to promote cognitive stimulation based on patient preferences	74/115 (64)	45/64 (70)	5/8 (63)	24/43 (56)	64/86 (74)	40/48 (83)	2/3 (67)	22/35 (63)
Ensure access to outside space where possible	71/115 (62)	46/64 (72)	6/8 (75)	19/43 (44)	59/87 (68)	35/48 (73)	2/3 (67)	22/36 (61)
Include the patient (when able) and family in the development of the weaning plan	66/107 (62)	33/60 (55)	6/8 (75)	27/39 (69)	56/87 (64)	28/48 (58)	3/4 (75)	25/35 (71)
Family participation in care and occupation tasks	71/113 (63)	38/62 (61)	4/8 (50)	29/43 (67)	52/84 (62)	29/47 (62)	2/3 (67)	21/34 (62)
Provide access to social support such as a social worker; or signposting to sources of support and advice agencies (patient and family)	62/110 (56)	32/62 (52)	4/7 (57)	26/41 (63)	50/87 (57)	25/48 (52)	3/4 (75)	22/35 (63)
Minimising visiting restrictions	59/115 (51)	32/63 (51)	4/8 (50)	23/44 (52)	48/84 (55)	26/47 (55)	2/3 (67)	23/35 (66)
Family presence or participation in rounds and planning meetings	58/114 (51)	29/63 (46)	4/8 (50)	25/43 (58)	43/86 (50)	17/48 (35)	3/3 (100)	23/35 (62)
Limit physiologic monitoring and routine blood tests	34/111 (31)	24/63 (38)	2/8 (20)	8/40 (20)	16/87 (18)	12/48 (25)	1/3 (33)	5/36 (14)

Supplementary material: Table 3

ACTIONABLE PROCESS OF CARE	ROUND ONE MEANS (SD)				ROUND TWO MEANS (SD)			
	Overall	Staff	Family	Patients	Overall	Staff	Family	Patients
Assess and manage symptoms (i.e., pain, breathlessness, tiredness, thirst)	8.1 (1.1)	8.2 (1)	8.5 (0.8)	7.8 (1.3)	8.5 (0.7)	8.7 (0.6)	8.4 (0.8)	8.5 (1)
Assess and treat psychological issues including anxiety, depression, and acute stress (with referral to psychiatry if required)	8.1 (1.1)	8 (1.2)	8.5 (1.1)	8.2 (1.1)	8.5 (0.9)	8.4 (1)	8.5 (0.7)	8.5 (1)
Ensure adequate nutritional support	8.2 (1)	8.5 (0.8)	8.3 (0.9)	7.8 (1.2)	8.5 (0.9)	8.7 (0.6)	8.3 (1.1)	8.7 (0.6)
Provide aids to enable patients to communicate (including method to access help) (referral to speech specialist if required)	8.1 (1.2)	8.2 (1)	8.3 (0.9)	7.8 (1.4)	8.5 (0.9)	8.7 (0.7)	8.3 (1.1)	8.5 (1)
Provide fundamental hygiene and elimination care	8 (1.2)	8.2 (1.1)	8.4 (0.7)	7.8 (1.4)	8.5 (0.8)	8.6 (0.7)	8.3 (0.6)	8.4 (0.9)
Regular physical rehabilitation (including early mobilisation) with setting and assessment of progress on weekly rehabilitation goals	8.2 (1.1)	8.3 (0.9)	8.3 (0.9)	7.9 (1.3)	8.5 (0.7)	8.6 (0.7)	7.7 (0.6)	8.4 (0.8)
Assess and treat non-respiratory muscle wasting	8.0 (1.1)	8.0 (1.1)	8.1 (1)	8.1 (1.1)	8.4 (0.8)	8.5 (0.8)	8.4 (0.8)	7.7 (0.6)
Assess swallowing function and establish safe return to normal drinking and eating (may require referral to speech and language therapy)	8.2 (1)	8.4 (0.9)	8.1 (1.1)	8 (1.1)	8.4 (0.9)	8.7 (0.7)	8.2 (1.1)	8 (1.7)
Use strategies to manage excess secretions or the inability to cough up secretions	8.1 (1)	8.2 (0.9)	8.4 (0.7)	7.9 (1.1)	8.4 (0.9)	8.5 (0.8)	8.3 (1)	8.3 (0.5)
Assess, prevent/treat complications associated with prolonged bed rest/ICU stay (i.e., pressure ulcers, constipation, DVTs)	7.9 (1.2)	8.1 (1)	8.4 (1.1)	7.6 (1.5)	8.3 (1)	8.4 (0.8)	8.1 (1.1)	8.7 (0.6)
Assess readiness to deflate the tracheostomy cuff, downsize or decannulate the tracheostomy as part of the weaning process	8 (1)	8.2 (0.9)	8.2 (0.8)	7.7 (1.1)	8.3 (1)	8.4 (1)	8.1 (1)	7.5 (0.7)
Enable appropriate and timely referral to palliative care	7.9 (1.3)	8 (1.3)	8.4 (0.9)	7.7 (1.3)	8.3 (1)	8.4 (1.1)	8.2 (1)	9 (0)
Review ongoing need for drugs used for sedation and decrease their use as able	7.9 (1.2)	8.3 (1)	8.1 (0.7)	7.4 (1.4)	8.3 (1)	8.5 (0.8)	7.9 (1.2)	7.7 (0.6)
Assess and treat respiratory muscle weakness	7.7 (1.3)	7.6 (1.3)	7.9 (0.8)	7.8 (1.3)	8.2 (1)	8.1 (1.1)	8.3 (0.8)	8 (0)
Involve the patient in bedside rounds and goals of care discussions as early as able	7.7 (1.4)	7.8 (1.2)	8.8 (0.5)	7.3 (1.6)	8.2 (1.1)	8.2 (1.1)	8.8 (0.5)	8.1 (1.2)
Minimize practices such as night-time light/noise that promote delirium	7.8 (1.3)	7.8 (1.1)	7.3 (1.8)	7.8 (1.4)	8.2 (1)	8.3 (0.9)	8.2 (1.2)	7.5 (0.6)
Provide regular oral care including toothbrushing	7.8 (1.2)	8.1 (1)	8 (0.9)	7.4 (1.4)	8.2 (1)	8.5 (0.8)	8 (1)	7.9 (1.2)
Assess and track ventilator weaning progress	7.9 (1.3)	8.1 (1.1)	7.2 (1.3)	7.7 (1.4)	8.1 (1.2)	8.3 (1.1)	8.1 (1.2)	6.3 (1.2)
Identify legal shared decision maker & ensure review/documentation of wishes, advanced directives or living will	7.7 (1.3)	7.8 (1.3)	7.8 (1.6)	7.6 (1.5)	8.1 (1)	8.2 (1)	7 (1.8)	8.2 (1)

Identify and use patient preferences for strategies to promote sleep	7.6 (1.4)	7.7 (1.2)	7.9 (1)	7.4 (1.6)	8 (1.1)	8.1 (1.1)	8 (1)	7.7 (1.2)
Appropriate and timely discharge planning (discuss and arrange safe transitions in care location)	7.7 (1.2)	7.8 (1.2)	7.4 (1.1)	7.6 (1.3)	7.9 (1.1)	7.9 (1.1)	7.8 (1.2)	8 (1)
Use a structured tool (i.e. weaning protocol or individualized weaning plan) to plan and guide weaning developed by the ICU team	7.6 (1.3)	7.8 (1.2)	6.8 (1.3)	7.3 (1.5)	7.8 (1.3)	8.1 (0.8)	5.3 (0.5)	7.7 (1)
Assess/prevent ocular disorders arising from incomplete eyelid closure	7.5 (1.4)	7.6 (1.3)	8.1 (0.9)	7.2 (1.5)	7.7 (1.1)	7.9 (0.9)	7.4 (1.4)	8 (1.4)
Conduct interprofessional team meetings to discuss patient and family-centered care plan	7.4 (1.4)	7.6 (1.3)	7.1 (1.5)	7.2 (1.5)	7.7 (1.3)	7.8 (1.3)	7.4 (1.3)	8.7 (0.6)
Enable continuity of care using shared interprofessional goals agreed with family and patient where possible	7.5 (1.3)	7.6 (1.2)	7.6 (1.3)	7.3 (1.4)	7.7 (1.2)	7.8 (1.2)	7.6 (1.2)	8.3 (1.2)
Provide regular proactive family meetings to set goals, devise a care plan, and share information	7.4 (1.5)	7.4 (1.4)	8.1 (1)	7.3 (1.7)	7.7 (1.3)	7.8 (1.3)	8.3 (1.2)	7.5 (1.2)
Enable access to activities (radio, tv, iPad) and personal possessions (including clothes) to prevent boredom, loneliness, and restore normality	7.1 (1.8)	7.7 (1.3)	6.9 (2.1)	6.1 (2)	7.6 (1.4)	8 (1.2)	7 (1.6)	7.3 (1.2)
Use patient diary to aid communication with and provide psychological support for patient and family	7.4 (1.5)	7.2 (1.5)	7.1 (1.1)	7.8 (1.4)	7.6 (1.4)	7.4 (1.5)	8 (1.2)	7.3 (1.5)
Assess endocrine function and treat endocrine dysfunction such as hyperglycemia and hypothyroidism	7.3 (1.6)	7.2 (1.5)	8.5 (0.8)	7 (1.8)	7.5 (1.3)	7.4 (1.2)	7.5 (1.5)	8 (1.4)
Preparing patient for more independence where possible, for example encouraging self-care activities, reducing observations (may require referral to occupational therapy)	7.3 (1.5)	7.7 (1.2)	6.8 (2.3)	6.9 (1.5)	7.5 (1.3)	7.8 (1.2)	7 (1)	7.2 (1.4)
Provide activities to promote cognitive stimulation based on patient preferences	7.2 (1.6)	7.5 (1.4)	7.6 (1.7)	6.7 (1.9)	7.5 (1.4)	7.8 (1.2)	7.7 (1.5)	7.1 (1.6)
De-escalate (including change to oral instead of IV drugs) or stop ICU pharmacotherapy and restart previous comorbidity pharmacotherapy	7.3 (1.4)	7.4 (1.3)	7 (1.7)	7 (1.5)	7.3 (1.3)	7.3 (1.2)	7.3 (1.3)	6 (1.7)
Ensure access to outside space where possible	7 (1.6)	7.4 (1.3)	7.5 (1.5)	6.5 (1.9)	7.3 (1.5)	7.5 (1.3)	6.9 (1.7)	7.3 (1.5)
Include the patient (when able) and family in the development of the weaning plan	7.1 (1.6)	6.9 (1.6)	7.3 (1.4)	7.3 (1.6)	7.3 (1.6)	7 (1.5)	8.3 (1.5)	7.5 (1.7)
Family participation in care and occupation tasks	7.1 (1.5)	7.1 (1.4)	7 (1.3)	7.3 (1.8)	7.2 (1.4)	7 (1.4)	7.4 (1.4)	7.7 (1.5)
Provide access to social support such as a social worker; or signposting to sources of support and advice agencies (patient and family)	6.9 (1.7)	6.8 (1.7)	6.7 (1.1)	7.1 (1.9)	7 (1.6)	6.7 (1.3)	7.1 (1.9)	7.8 (1.5)
Minimising visiting restrictions	6.5 (2.1)	6.5 (1.9)	6.5 (2.1)	6.4 (2.5)	6.8 (2.1)	6.9 (1.9)	7.3 (1.5)	6.7 (2.4)
Family presence or participation in rounds and planning meetings	6.6 (1.5)	6.3 (1.4)	7 (1.5)	6.8 (1.7)	6.6 (1.3)	6.1 (1.1)	7.2 (1.4)	8.3 (0.6)
Limit physiologic monitoring and routine blood tests	5.8 (1.8)	6.2 (1.6)	5.6 (2.1)	5.2 (2)	5.6 (1.5)	6 (1.2)	5.7 (1.5)	5 (1.7)

Supplementary information: Figure 1

Prolonged ICU stay care plan – how each item was included

TOPIC	ITEM	METHOD OF INCLUSION
1. INVOLVING PATIENT AND FAMILY IN GOAL SETTING AND DECISION MAKING	a. Involve patient in bedside rounds/goals of care discussions as early as able	Rated > 8 in Delphi survey
	b. Provide regular proactive family meetings to set goals, devise a care plan, and share information. Last family update: __________	Rated for inclusion in Consensus meeting
	c. Identify legal shared decision maker & ensure review/documentation of wishes, advanced directives or living will	Rated > 8 in Delphi survey
2. PROVIDE AIDS TO ENABLE PATIENT TO COMMUNICATE (INCLUDE METHOD TO ACCESS HELP)		Rated > 8 in Delphi survey
3. PROMOTE PHYSICAL COMFORT AND MINIMIZE COMPLICATIONS	a. Prevent/treat complications associated with prolonged bed rest/ICU stay (i.e., pressure ulcers, constipation, DVTs)	Rated > 8 in Delphi survey
	b. Provide regular oral care including toothbrushing	Rated > 8 in Delphi survey
	c. Provide fundamental hygiene and elimination care	Rated > 8 in Delphi survey
	d. Assess/manage symptoms (pain, breathlessness, tiredness, thirst)	Rated > 8 in Delphi survey
	e. Ensure adequate nutritional support	Rated > 8 in Delphi survey
4. PROMOTE SELF-CARE AND RESTORE NORMALCY	a. Enable access to activities (radio, tv, iPad) and personal possessions (including clothes) to prevent boredom, delirium, loneliness, and restore normality - might require a referral to Occupational Therapy	MERGED ITEM – suggested in consensus meeting
	b. Use patient diary to aid communication with and provide psychological support for patient and family	Included by research team due to evidence for use in UK ICUs
5. OPTIMIZE VENTILATOR WEANING	a. Track and guide ventilator weaning using a structured tool (protocol/individualized weaning plan)	MERGED ITEM: Assess and track ventilatory weaning progress rated >8 in Delphi survey

		Use a structured tool – included by research team due to evidence for use in ICU
	b. Assess causes of weaning failure, including respiratory muscle weakness (provide respiratory muscle training as appropriate) and endocrine function	MERGED ITEM: Assess and treat respiratory muscle weakness rated >8 in Delphi survey, and review of endocrine function included due to evidence base.
	c. Assess readiness to deflate the tracheostomy cuff, downsize or decannulate the tracheostomy as part of the weaning process	Rated > 8 in Delphi survey
	d. Use strategies to manage excess secretions/inability to cough up secretions	Rated > 8 in Delphi survey
6. OPTIMIZE PHYSICAL RECOVERY	a. Regular physical rehabilitation (including early mobilisation) with setting and assessment of progress on weekly rehabilitation goals)	Rated > 8 in Delphi survey
	b. Assess and treat non-respiratory muscle wasting	Rated > 8 in Delphi survey
7. ASSESS SWALLOWING FUNCTION AND ESTABLISH SAFE RETURN TO NORMAL DRINKING AND EATING<br>- might require a referral to Speech and Language Therapy		Rated > 8 in Delphi survey
8. DE-ESCALATE (INCLUDING CHANGE TO ORAL INSTEAD OF IV DRUGS) OR STOP ICU PHARMACOTHERAPY AND RESTART PREVIOUS COMORBIDITY PHARMACOTHERAPY)		Included by research team due to evidence base for poor implementation in practice
9. ASSESS AND TREAT PSYCHOLOGICAL ISSUES INCLUDING ANXIETY, DEPRESSION, AND ACUTE STRESS<br>- might require a referral to Psychology		Rated > 8 in Delphi survey
10. MINIMIZE DELIRIUM RISK	a. Use patient preferences for strategies to promote sleep, including reducing night-time light/noise	MERGED ITEM -both items rated > 8 in Delphi survey
	b. Review ongoing need for sedation and decrease as able	Rated > 8 in Delphi survey
11. ENSURE CONTINUITY IN CARE	a. Conduct interprofessional team meetings to discuss patient- and family-centered care plan and setting interprofessional goals	MERGED ITEM – suggested in consensus meeting
	b. Appropriate and timely discharge planning (discuss and arrange safe transitions in care location)	Rated for inclusion in Consensus meeting
	c. Enable appropriate and timely referral to palliative care	Rated > 8 in Delphi survey
